# The Impact of Wages on Care Home Quality in England

**DOI:** 10.1093/geront/gnad032

**Published:** 2023-03-25

**Authors:** Stephen Allan, Florin Vadean

**Affiliations:** Personal Social Services Research Unit (PSSRU), University of Kent, Canterbury, UK; Personal Social Services Research Unit (PSSRU), University of Kent, Canterbury, UK

**Keywords:** Care homes, Long-term care, Nursing homes, Service ratings, Staff

## Abstract

**Background and Objectives:**

In many countries, a large proportion of long-term care staff are paid at, or near, minimum wage, leading to concerns of negative effects on care outcomes. This study analyzed the effect of staff wages on care home quality ratings in England.

**Research Design and Methods:**

A national staffing database of long-term care providers was matched with local-area information on needs and supply to construct a 3-year panel (2016–2018) of English care home observations. Using multiple imputation methods to address missing data provided a data set of 12,055 observations of 5,556 care facilities (both residential and nursing homes). We analyzed the effect of the facility-level average hourly wage of care staff on national regulator quality ratings. A measure of the impact of exogenous changes in the national minimum wage on care facilities was used as an instrument for wage.

**Results:**

We find that wages positively affect care home quality ratings. Other things equal, a 10% increase in the average hourly wage of direct care workers would lead to a 7.1% increase in the likelihood that a care home will have a high-quality rating. The wage effect on quality was significant when controlling for staff skill mix, measured as the share of registered nurses in nursing home staff.

**Discussion and Implications:**

This study provides important evidence of the positive impact that staff pay can have on the quality of long-term care. Our finding has important implications for appropriate levels of pay and the funding of long-term care.

Staff play a crucial role in the aspects of quality of care provided in a care home (often called nursing homes, residential aged care facilities, skilled nursing facilities, or aged care homes) and form relationships with those they care for (Donabedian, 1988; Hussein, 2017; Lucas et al., 2007; Malley & Fernandez, 2010). There are a large number of empirical studies directly analyzing how staffing affects long-term care (LTC) providers’ performance. These have examined the relationship between quality and staffing levels and skill mix (e.g., Boscart et al., 2018; Dellefield et al., 2015; Konetzka et al., 2008) through staff turnover and retention (e.g., Allan & Vadean, 2021; Castle, 2021; Castle et al., 2020; Huang & Bowblis, 2019).

At the same time, research on staff wages in LTC has found that minimum wage policy had a positive impact on wages and (depending on context) either no or small positive effects on employment (Machin & Wilson, 2004; McHenry & Mellor, 2022; Ruffini, 2022; Vadean & Allan, 2021). Similarly, research evidence on local LTC wage policy initiatives (e.g., state Medicaid wage pass-through policies) found they improved staff wages (Baughman & Smith, 2010) and employment (Feng et al., 2010; Foster & Lee, 2015; Wu et al., 2021). Moreover, higher wages have been shown to improve LTC staff retention (Baughman & Smith, 2012; Gleason & Miller, 2021; Kennedy et al., 2021; Powers & Powers, 2010; Vadean & Saloniki, 2023; Wiener, 2009).

There is some research into the effect of wages on LTC quality in particular. Cawley et al. (2006) found that higher wages in U.S. nursing homes led to factor substitution and lowered elements of care quality. Conversely, state Medicaid wage pass-through policies significantly improved nursing home resident outcomes (Foster & Lee, 2015), and nursing homes facing a higher minimum wage have higher quality, including reduced violations and fewer preventable conditions and deaths (Ruffini, 2022). In England, the introduction of a national minimum wage reduced the ratio of supervisory staff to care staff in care homes, indicating that the cost of higher wages was offset by reduced monitoring costs and a link between pay and productivity (Georgiadis, 2013). This study adds to this existing literature by assessing the effect of wages on facility-level quality in England using data from a national staffing database of LTC providers for the years 2016–2018.

## English Care Homes

The English care homes market for those of older age consists of around 11,000 homes that are registered with the national health and LTC regulator, the Care Quality Commission (CQC). These homes include both nursing homes and residential homes offering personal care only. The latter are similar to assisted living facilities in the United States (Allan & Vadean, 2021). Care homes must adhere to fundamental standards of care which providers should not fall under (Care Quality Commission, 2015a). Care homes are predominantly in the independent sector, with the vast majority of homes run as for profit. The market can be considered very competitive, with only a small number of larger providers (Forder & Allan, 2014). Similar to the United States, demand comes from two main sources, roughly split equally in terms of overall numbers: private payers (self-funders) and public funding to support those who cannot afford to (fully) pay for themselves and have a certain level of LTC needs.

In England and elsewhere, working in care homes, particularly as a care worker (i.e., nurse aide or nursing assistant in the United States), has a negative perception, as the role is seen as low paid, low skilled (i.e., little or no education required), and with little in the way of career progression (Hussein, 2017; Organisation for Economic Co-operation and Development, 2020; Rajamohan et al., 2019). Staff tend to be low paid, often at minimum wage (OECD, 2020; Skills for Care, 2021). However, those working in LTC tend to do so for inherent reasons, have high levels of skills acquired informally (Hussein, 2017), and may be paid a low wage because of their caring motive (Barron & West, 2013; Wagner et al., 2021).

Overall, given the similarity between the market system of English care homes and the low pay of staff when compared to other LTC systems across the world, including the United States, findings on how wage affects care quality in England will have wider implications for LTC internationally.

## Conceptual Framework

**Figure 1.**
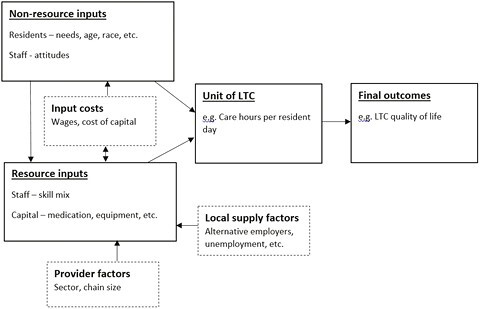
Production of welfare model, adapted from Knapp (1984). LTC = long-term care.

We analyzed the quality of LTC to an individual through the production of welfare approach (Knapp, 1984; Malley & Fernandez, 2010). This approach directly assesses how an LTC service can influence the welfare of individuals (see Figure 1). In particular, the inputs into services, including staff and equipment, and nonresource factors, such as care home residents’ needs and attitudes of staff, will influence the amount of service produced and the quality of this service. Ultimately, these factors will affect the well-being of residents, be that measured in terms of clinical outcomes, for example, bed sores, or overall LTC quality of life (Forder et al., 2018).

The production of welfare approach therefore directly links staff, including their cost and attitudes, to production outcomes in care homes. If wages and other intrinsic and extrinsic benefits to working with an organization have a positive effect on the level of staff ability and attitudes then this will influence the quality of care. A theoretical model through which wages can influence quality is the efficiency wages model (Salop, 1979; Stiglitz, 1982). In this model, a higher wage leads to higher productivity through either improving the quality of job applicants, making it more difficult to move jobs (reducing turnover), or incentivizing staff to improve productivity (Akerlof & Yellen, 1986; Katz, 1986; Krueger, 1991).

For care homes, given their fixed size (in the short run), we might expect that productivity rises would be seen through improvements in the quality of care, providing a better quality of life and/or increased life expectancy. As such, we expected an increase in the (average) wage paid to direct care workers to lead to an increase in a facility’s care quality.

Figure 1 also shows additional ways in which the wage–quality relationship can be explained in the production of welfare approach, which needs to be controlled when analyzing the wage–quality relationship in care homes. Skill mix in a care home will be an important factor (Dellefield et al., 2015). Increased ability due to training and education will increase productivity and the wage paid to an employee (Becker, 1993; Dearden et al., 2006; Parent, 1999). Moreover, the sector of the facility, the needs levels of the residents, and the payment system faced may influence the wage–quality relationship (Borjas et al., 1983; Huang & Bowblis, 2020; Malley & Fernandez, 2010). For example, residents with higher care needs will necessarily require a greater level of services to be provided. These residents will therefore have their quality of life affected to a greater extent by the provision of services (Forder et al., 2018). This would create an upward bias in the size of the effect on quality by factors influencing service production, including wages.

Factor substitution could also affect the relationship between wage and quality. A higher wage for direct care staff can increase their cost relative to other factors of production, such as alternative staffing or other care inputs, for example, increased use of medication, which are seen as lower quality (Cawley et al., 2006; Georgiadis, 2013; Zinn, 1993). We would expect the wage–quality relationship to be negative in this instance, applying a downward bias to the observed wage–quality relationship.

Overall, the conceptual framework informed the statistical model used to analyze the effect of wages on the quality of English care homes, taking into account potential confounding explanations. The hypothesis examined was that a higher average care worker wage had a positive effect on care home quality.

## Method

### Data Sources

We used the Adult Social Care Workforce Data Set (ASC-WDS) provided by Skills for Care, which is a database of provider staffing at employee and provider levels and the main source of LTC workforce intelligence for England (Skills for Care, 2021). Skills for Care matched an anonymized provider-level database to quality indicators (see later) as of October for the years 2016–2018 for our analysis. We used the employee-level database to generate wage and other individual characteristics, which we then averaged and matched to the provider-level database. Finally, we obtained local-area characteristics from publically available data sets from the Office for National Statistics (benefits data) and Land Registry (house prices) which we matched to care home observations at postcode district level (the first half of a U.K. postcode, e.g., SW1, *n* = 2,302).

For each wave, we only included care homes that had updated their information in the prior 6 months. This created an unbalanced panel of 5,556 independent sector care homes observed over the 3 years, providing 12,055 care home observations in total, with 2,541 homes being present in the data for all 3 years. With around 4,000 care home observations in each year, the data included more than a third of all English care homes for older people.

### Wage

We assessed the effect of average direct care worker wage on CQC quality ratings of English care homes. Care workers, much like nurse aides or nursing assistants in the United States, deliver the vast majority of direct care to residents (Skills for Care, 2021). Their average hourly wage at the provider level was calculated using wage data from the employee-level database and was weighted for inflation to October 2018 prices.

### Covariates

Following the conceptual framework above, we included a number of controls at care home and local-area levels which were likely to influence quality and wage and are typically included in the U.S. literature on nursing home quality (e.g., Castle, 2021). At care home level, we used the following variables available in the ASC-WDS: care home sector (for profit or not for profit), type (residential or nursing), size (total number of beds), a measure of financial status (occupancy rate, calculated as reported utilization to beds), and a proxy measure of resident needs/cognitive impairment (if the care home supported those living with dementia). Further, at staff level, we controlled for total staff, staff to service user ratio, the proportion of supervisors to care workers, the proportion of staff that was female, the proportion of staff that has received dementia training, and the proportion that has received training for person-centered care or dignity. For models only including nursing homes, we included a further indicator of skills mix: the percentage of staff that was registered nurses.

At the local-area level, on the supply side, we included an inverse measure of care home competition, a distance-weighted Herfindahl–Hirschman Index, and the percentage of females that were claiming Job Seeker’s Allowance, an unemployment benefit. On the demand side, given there was no information on resident funding sources, we included proxy measures of self-funding level: the percentage of the older population claiming Pension Credit (an income-based benefit and an inverse indicator of self-funding) and the average house price (Forder & Allan, 2014). For local-area care needs, we included the share of older population claiming Attendance Allowance (a needs-based benefit). Finally, we included binary variables indicating year of observation and region of England. The former was included to capture any changes over time not included in the covariates, for example, changes to the inspection and rating system, and the latter to capture potential regional differences in local care policy, commissioning, and care markets.

### Quality

We measured quality using the CQC’s quality rating system, which looks to assess people’s experiences of care (Care Quality Commission, 2017). The rating is centered on an inspection of the care home and constructed around five key questions asking if the home is Responsive to people’s needs, Safe, Caring, Effective, and Well-led. Key lines of enquiry are used to consistently assess the five questions, and homes are given a rating for each of the five questions of either “Inadequate,” “Requires improvement,” “Good,” or “Outstanding.” The overall rating for a care home uses the same four levels and is determined from consistent aggregation of the ratings for the five key questions (Care Quality Commission, 2015b). Because of the low number of homes rated as “Inadequate” and “Outstanding,” for the analysis, we used a binary variable indicating high quality (0 if a home was rated as “Inadequate” or “Requires improvement” and 1 if a home was rated as “Good” or “Outstanding”). Care homes moved between ratings over time, including between the binary indicator of quality used in the analysis (Care Quality Commission, 2017).

### Model Specification

From the conceptual framework of quality developed above and given the measure of quality available we used a latent probability model of quality in the analysis, subject to making a level of profit to remain in business (Allan & Forder, 2015). Consider that each facility has an actual level of quality, $q^{a}$, which can be expressed in the following manner:


$$\begin{array}{l} q_{it}^{a}=\alpha_{1}+\alpha_{2}w_{it}+\alpha_{3}X_{it}+v_{it}, \end{array}$$

where quality depends on staff wages, w, the vector $X_{it}$ of other exogenous care home and local demand and supply factors described above, and a random error. Observed quality of care homes at time of inspection, $q^{o}$, will measure actual quality with some level of error and so we modeled that the inspection outcome, that is, the rating, depended on the following decision rule:


$$\begin{array}{l} \begin{array}{lll} & q_{it}^{o}=0\;\;if\;q_{it}^{a}<0\; & q_{it}^{o}=1\;\;if\;q_{it}^{a}\;\;\geq \;0,\; \end{array} \end{array}$$

where homes rated as $q^{o}=0$ had a quality rating of “Inadequate” or “Requires improvement” and homes rated as $q^{o}=1$ a quality rating of “Good” or “Outstanding.”

### Statistical Methods

The data set contained missing information. We have shown elsewhere that the data in ASC-WDS are not missing completely at random, that is, the likelihood of a facility having missing data is correlated with observable facility data such as sector and size, and therefore analysis using only complete cases would yield biased findings (Allan & Vadean, 2021). Therefore, we assumed that the data were missing at random, that is, independent of unobservable data, and used multiple imputations generated from ordered logit (quality) and predictive mean matching models (*n* = 50) to create predicted values for missing data (White et al., 2011).

We also controlled for the likelihood that wage is endogenous in a model of facility quality due to an omitted variable, for example, unobserved staff skills or work values, or simultaneity bias, for example, higher wages rewarded for a quality improvement. A common measure used in the minimum wage literature, which has also been used as an instrument for wage, is the impact of exogenous national minimum wage increases (Card & Krueger, 1994; Cawley et al., 2006; Draca et al., 2011; Machin & Wilson, 2004). Specifically, we measured the proportion of workers employed by provider i that were being paid less than the future National Living Wage rate, and assumed it had no direct effect on facility quality. The National Living Wage increased in each year of our analysis, from £7.20 per hour in 2016 for those aged 25 and over, to £7.50 in April 2017, £7.83 in April 2018, and £8.21 in April 2019. We assessed for the strength and exogeneity of the instrument using relevant tests (Wooldridge, 2010).

Given the panel nature of the data and the use of an instrumental variable, we estimated a linear probability model (LPM) of quality ratings using OLS (Wooldridge, 2010):


$$\begin{array}{l} P\left(q_{it}^{0}=1\right)=\;\beta_{1}+\beta_{2}w_{it}+\beta_{3}X_{it}+\varepsilon_{it} \end{array}$$

We estimated this model first using a pooled cross-section specification with wage treated as exogenous before estimating pooled and random effects LPMs of quality with wage treated as endogenous. The random effects specification was shown to be appropriate using a Mundlak test (Mundlak, 1978). We then further estimated the random effect LPM of quality for nursing homes only, where a further measure of skill mix, the percentage of staff that was registered nurses, could be included. Finally, for comparison, we ran the same models using the complete case observations only. Stata 16 was used for the analysis, specifically using *reg*, *ivreg2*, and *xtivreg* commands, and we clustered standard errors by care home.

## Results

There were 12,055 homes in the overall sample, 4,062 in 2016, 4,022 in 2017, and 3,971 in 2018. Summary statistics for these homes are presented in Table 1. The majority of facilities were for profit, residential homes providing dementia care, and the average size was 40 beds. The quality of the facilities improved on average over the 3 years, with four in five rated as “Good” or “Outstanding” in 2018. Average hourly wage increased by £0.24 per hour (3.1%), but there was little indication in the data of changes to staffing levels, with the total staff size, and the ratio of care staff to service users and supervisory staff not altering greatly. However, for nursing homes, there was a 2.6 percentage point fall in the proportion of staff that was registered nurses, suggesting a decrease in skill mix.

**Table 1.** Summary Statistics for All Facilities

Variable	2016		2017		2018	
	*n*	Mean	*n*	Mean	*n*	Mean
Facility structure						
Not for profit (%)	4,062	12.2	4,022	13.0	3,971	10.1
Facility is nursing home (%)	4,062	37.2	4,022	38.2	3,971	39.6
Facility provides dementia care (%)	4,062	69.8	4,022	70.1	3,971	70.3
Size	4,062	40.2 (24.1)	4,022	40.2 (24.4)	3,971	41.6 24.0)
Occupancy rate	4,062	92.4 (13.2)	4,022	91.7 (13.6)	3,971	91.2 (14.4)
Herfindahl−Hirschman Index	4,057	0.056 (0.074)	4,018	0.055 (0.072)	3,969	0.056 (0.074)
Quality						
“Good”/“Outstanding” (%)	3,442	69.7	3,809	76.7	3,755	79.6
Staffing						
Staff size	4,062	44.5 (29.6)	4,022	44.1 (29.8)	3,971	45.8 (30.1)
Direct care staff to service user ratio	4,062	0.90 (0.43)	4,022	0.92 (0.45)	3,971	0.90 (0.41)
Supervisor to direct care staff ratio	4,042	0.12 (0.09)	3,988	0.12 (0.09)	3,956	0.11 (0.09)
Registered nurse (%)^a^	1,043	10.2	1,090	8.5	1,177	7.6
Female staff (%)	2,912	86.4	2,941	86.5	2,939	86.8
Training incidence						
Dementia (%)	2,916	28.0	2,947	28.4	2,949	27.9
Dignity/person-centered care	2,916	12.1	2,947	13.2	2,949	12.8
Wage						
Direct care worker hourly wage (£2018)	2,556	7.75 (0.70)	2,559	7.82 (0.61)	2,590	7.99 (0.61)
Staff below future minimum wage (%)	2,264	47.9	2,297	51.2	2,317	53.3
Local-area controls						
Female job seeker’s allowance (%)	4,062	0.85	4,022	0.8	3,971	0.6
Attendance allowance (%)	4,062	12.5	4,022	12.3	3,971	12.3
Pension credit (%)	4,062	16.4	4,022	15.1	3,971	14.1
Average house price (£2018)	4,062	211,817 (111,066)	4,021	211,419 (115,660)	3,971	208,193 (110,710)

*Note:* Mean and standard deviation are presented for continuous variables, percentages presented for binary variables.

^a^Registered nurse percentage is for nursing homes only. Controls also included for year (2016, 2017, and 2018) and region of England (East of England, East Midlands, London, North East, North West, South East, South West, West Midlands and Yorkshire, and Humberside).

The estimation results are presented in Table 2. The first three columns present the estimates of the model of quality for all facilities whereas the latter two columns present the results when the model is estimated for nursing homes only. Pooled cross-section specifications are presented in the first two columns, whereas the latter three columns take into account the panel nature of the data. Direct care worker’s average hourly wage is treated as endogenous in all but the first column.

**Table 2.** Results of Estimating Model of Facility Quality

Variable	(1)	(2)	(3)	(4)	(5)
	MI CS All facilities	MI CSIV All facilities	MI REIV All facilities	MI REIV NH only	MI REIV NH with skill mix
Facility structure					
Not for profit	0.056**	0.020	0.027	−0.009	−0.003
	(0.015)	(0.016)	(0.016)	(0.035)	(0.035)
Nursing home	−0.038**	−0.036**	−0.037**		
	(0.012)	(0.012)	(0.012)		
Dementia care	−0.044**	−0.039**	−0.043**	−0.029	−0.025
	(0.011)	(0.011)	(0.011)	(0.017)	(0.018)
Size (log)	0.010	0.007	0.001	−0.021	−0.017
	(0.014)	(0.014)	(0.013)	(0.022)	(0.022)
Occupancy rate	0.004**	0.004**	0.004**	0.004**	0.004**
	(0.0004)	(0.0004)	(0.0004)	(0.001)	(0.001)
Competition (Herfindahl−Hirschman Index)	0.256**	0.245**	0.233**	0.416**	0.410**
	(0.062)	(0.063)	(0.063)	(0.105)	(0.104)
Staffing					
Total staff (log)	−0.028**	−0.025	−0.018	−0.013	−0.016
	(0.013)	(0.014)	(0.013)	(0.025)	(0.025)
Direct care staff to resident ratio	0.190**	0.179**	0.175**	0.153	0.156
	(0.047)	(0.048)	(0.047)	(0.086)	(0.086)
Direct care staff to resident ratio squared	−0.045**	−0.047**	−0.048**	−0.042	−0.040
	(0.017)	(0.017)	(0.017)	(0.032)	(0.032)
Supervisor to direct care staff ratio	0.124*	0.111	0.111	0.134	0.139
	(0.058)	(0.059)	(0.060)	(0.129)	(0.129)
Female staff (%)	0.001	0.001	0.001	0.001	0.001
	(0.0006)	(0.0006)	(0.0006)	(0.001)	(0.001)
Registered nurse (%)					0.144
					(0.109)
Training incidence					
Dementia trained staff (%)	0.001**	0.001**	0.001**	0.001*	0.001*
	(0.0002)	(0.0002)	(0.0002)	(0.004)	(0.0003)
Dignity/PCC trained staff (%)	0.0002	0.0002	0.0001	−0.000005	−0.0001
	(0.0002)	(0.0002)	(0.0003)	(0.0005)	(0.0005)
Wage					
Direct care staff hourly wage (£2018) (log)	0.207**	0.785**	0.705**	0.897**	0.849**
	(0.075)	(0.125)	(0.132)	(0.235)	(0.233)
Local-area controls	YES	YES	YES	YES	YES
Year	YES	YES	YES	YES	YES
Region	YES	YES	YES	YES	YES
Observations	12,055	12,055	12,055	4,617	4,617
Number of facilities	5,556	5,556	5,556	2,130	2,130
Imputations	50	50	50	50	50
Average RVI	0.143	0.153	0.187	0.168	0.182
Largest FMI	0.385	0.414	0.484	0.447	0.444

*Notes:* CS = cross-section; FMI = fraction of missing information; IV = instrumental variable; MI = multiple imputation; NH = nursing homes; PCC = person-centered care; REIV = random effects instrumental variable; RVI = relative variance inflation. Robust standard errors in parentheses (clustered at facility level). All models estimated are linear probability models using OLS. Local-area controls are Job Seeker’s Allowance uptake, pension credit uptake, attendance allowance uptake, and average house price (log).

*p < .05. **p < .01.

The impact of hourly wage on quality ratings is significant and positive across all estimations, confirming the main hypothesis. Based on the estimation presented in column 3, a 10% rise in average hourly wage would increase the likelihood of a care home being rated “Good” or “Outstanding” by 7.1%, other things equal. As an assessment of the robustness of this finding, the final two columns show the results from estimating the model of care quality for nursing homes only. The results in column 4 are generally consistent with those when estimating the model for all facilities, with a higher wage effect found. In the final column, we added a further measure of skill mix to the model, the percentage of staff that were registered nurses, and the wage effect reduced only modestly. The findings also did not change markedly when we only used the complete case home observations in the model of facility quality, with some upward bias in effect size observed (see Supplementary Table 1).

The use of instrumental variables depends on the quality of the instrument(s) used. Overall, we found evidence of the endogeneity of care workers’ average hourly wage, that the instrument was strong and was not overidentified or correlated with quality ratings in an unidentified way (Towers et al., 2021). As such, the results indicate that there was a substantial downward bias of the wage effect on quality.

In terms of other findings, nursing homes (as compared to residential homes), homes that cared for residents living with dementia, and homes facing greater competition had significantly lower quality. Staffing-wise, the more direct care staff to residents the higher the likelihood of a high-quality rating, subject to diminishing marginal returns. Dementia training incidence had a significant direct effect on the likelihood of high quality. This could indicate a positive effect of training to productivity in addition to increased human capital or could be an indication that training levels amongst staff are seen as a “signal” by the regulator for better quality providers.

## Discussion

Staff play a vital role in the delivery of LTC services. Although there is a large existing literature on the effects of staffing on quality (e.g., Backhaus et al., 2014) and the impact of minimum wage on staffing (e.g., McHenry & Mellor, 2022; Vadean & Allan, 2021), the evidence as to the impact that staff remuneration has on care quality has mainly been at the policy level (Feng et al., 2010; Foster & Lee, 2015). A recent exception is Ruffini (2022), who analyzed the effects of minimum wages in the U.S. nursing home industry, finding evidence of significant improvements in staff retention and resident outcomes for those homes with higher staff wages. Our analysis added to this research by assessing the effect of wages on the quality of LTC facilities (both residential and nursing) in England using a national staffing database for 2016–2018, which included staff wage data. LTC firms made decisions on staff pay, subject to the conditions they faced, which included an annually uprated national minimum wage. We found that facilities that paid a higher average wage to care staff had higher quality. We have controlled for a large number of observed factors that are likely to affect the wage–quality relationship, such as needs, sector, financial status, resident wealth, and staff skills. We have also controlled for the endogeneity of wage. Overall, our finding complements the evidence for the United States and extends the existing literature for English LTC facilities.

The production of welfare approach provided a theoretical model from which labor inputs, including staff attitudes, can influence resident outcomes, and efficiency wage theory offers an explanation for how an increased wage can improve productivity. From this, we hypothesized that an increased average wage would cause increased productivity from workers due to improved attitudes and less shirking, improving overall facility quality. Our findings support this hypothesis, but we have been unable to fully assess what is driving the quality improvement. We found that the wage effect was stronger in nursing homes (as compared to residential homes), where, given higher levels of needs, the potential to shirk would be stronger. This adds to the findings of Ruffini (2022), who showed that the positive association between wages and quality was unlikely due to poor-performing firms exiting the market, or changes in the characteristics of patients and staff. However, further research is required to assess why care staff wage affects quality.

The finding that higher staff wages can improve quality is important for those people who are looking (or maybe looking in the future) for a care home place. How staff are treated in their job roles, including their pay, is an important factor that should be considered. There are also implications for LTC providers. If the quality improvement increased revenue then there is incentive for providers to improve the wages of their staff (Ruffini, 2022; Weech-Maldanado et al., 2019). However, although there is evidence of at least some level of firm market power, that is, an ability to set prices (Forder, 2000; Mukamel & Spector, 2002; Nyman, 1989), both in England and the United States, public funding has a dominant role in the demand for care, supporting those unable to (fully) fund themselves. Budget constraints and increased demand for care support have led to downward pressure on prices paid for publicly funded care, which are generally cross-subsidized with higher prices to the private pay segment of the market (Allan et al., 2021; Forder & Allan, 2014; Grabowski, 2004). The ability of providers to raise prices—to at least a large part of the market—is therefore open to question.

In England during the period 2016–2018, the National Living Wage was introduced and pushed up the minimum wage in the economy by more than 16%. If price rises could not cover this cost in full, providers in the LTC sector will likely have suffered a cut in profits, substituted factors of production, or some combination of the three. There is evidence of factor substitution in the United States (Cawley et al., 2006; Zinn, 1993). However, given that care home quality in England increased over the timeframe analyzed, we might rule out that factor substitution dominated any adjustment by providers, albeit we did find evidence of at least some reduced skill mix in English nursing homes.

As staffing accounts for a large proportion of costs in a care home, we might therefore tentatively expect that profits will have shrunk. Although Ruffini (2022) found that U.S. nursing homes facing higher minimum wage levels offset the higher costs through increased focus on the self-pay portion of the market and charging higher prices to these residents, the introduction of the national minimum wage for England in 1999 lowered care home profits (Draca et al., 2011). This could have important implications for the sustainability of the LTC sector, if current levels of public funding did not keep pace with increasing production costs.

Also, although we have found that care homes that paid higher average wage had better quality, efficiency wages can also be prevalent at the industry level, reducing turnover compared to other industries (Krueger and Summers, 1988). Job vacancies and staff turnover are ongoing issues in the LTC sector (OECD, 2020), and pay is often lower in LTC compared to similar roles in health care (Wagner et al., 2021). Research for the United States has shown that state Medicaid wage pass-through policies have been successful at raising both wages (Baughman & Smith, 2010) and elements of quality including staff hours per resident day and rate of pressure ulcer worsening (Feng et al., 2010; Foster & Lee, 2015). In the U.K., there is evidence in support of higher wages improving productivity in the care home sector (Georgiadis, 2013). There are also positive effects on staff retention from higher wages (Ruffini, 2022; Vadean & Saloniki, 2023; Wiener et al., 2009). Given the importance of staffing in LTC, higher pay to improve recruitment and reduce turnover would help to ensure high quality (Allan & Vadean, 2021; Castle et al., 2020; Konetzka et al., 2008). Finally, the benefits of increased cost to raise wages would also include reduced use of health services from better resident outcomes. Overall, however, as earlier, appropriate public funding would be required to allow all LTC providers to increase staff pay.

### Limitations

There were a number of limitations to this study. First, there may be unobserved factors that bias the main finding. Other than broad indicators by facility, the needs levels of residents were not known. Also, not known were residents’ socioeconomic characteristics such as age, gender, ethnic background, and income. As outlined earlier, those that fund their own care privately are known to pay higher prices for their care, and, for example, there is evidence of differences in care quality by ethnic background (Smith et al., 2007). We did include controls for needs and income at the local-area level and the statistical methods employed took into account time-invariant unobserved factors. Linked to this, a caveat to our argument of the wage effect on quality being at the provider level is that we could not control for any variation in local LTC policy over the period analyzed that may have influenced the relationship between wage and quality, for example, wage pass-through policies.

The instrument used in the analysis, changes to national minimum wage, was assumed to be exogenous of quality. However, this assumption may not hold if National Living Wage increases had an effect on employment levels, that is, a reduction in staff. At a descriptive level, we found minimal evidence of staff alterations, other than for the number of registered nurses employed by nursing homes, and we controlled for staffing in the analysis, including registered nurse percentage for nursing homes. Further, there is little evidence that the recent changes to the minimum wage in England reduced employment in the LTC sector (Vadean & Allan, 2021).

The measure of quality used in the analysis was the quality rating of the facility. Therefore, unlike Ruffini (2022) for the United States, this analysis was unable to directly assess whether increased wages brought improved outcomes for English care home residents. However, importantly, the quality rating system of care homes in England looks to assess people’s experiences of care and there is evidence of a significant positive relationship between the overall rating of a care home and residents’ quality of life (Towers et al., 2019, 2021). We would therefore expect that English care home resident outcomes were improved by higher wages. Finally, we could not assess any effects on health outcomes from improved quality.

Given these limitations, further research is needed to quantify the savings that higher pay can generate from improved quality of care and reduced health care use. This will help inform the appropriate distribution of public funds between health and LTC. For England in particular research is also required to assess the effect of wages on resident outcomes and the impact of local wage policy on staffing and care quality.

## Conclusion

Overall, the finding that higher pay is linked to better care quality is important for the delivery of LTC internationally, where staff are paid low wages and there are high levels of staff turnover. Promoting the value of a job in the delivery of LTC through higher wages, particularly above jobs viewed as comparable in alternative industries, such as in hospitality and retail, could add value to the industry through increasing staff quality, reducing staff turnover, and, ultimately, improving resident outcomes.

## Supplementary Material

gnad032_suppl_Supplementary_Material

## Data Availability

The study was part of a wider project which was preregistered with NIHR (https://fundingawards.nihr.ac.uk/award/15/144/51). The data used for this analysis were obtained under a data sharing agreement with Skills for Care. The code used for the analysis is available upon request from the authors.
