# Supplementary material for: The Impact of Wages on Care Home Quality in England
Source: Gerontologist. 2023 Mar 25;63(9):1428–36. doi: 10.1093/geront/gnad032 (PMC10581380; doi:10.1093/geront/gnad032)
Supplement: gnad032_suppl_Supplementary_Material [file gnad032_suppl_supplementary_material.docx]

**Online Supplementary Material**

Supplementary Table 1: Results of estimating model of facility quality, complete cases

|  | (1) | (2) | (3) | (4) | (5) |
| --- | --- | --- | --- | --- | --- |
| VARIABLES | CS  All facilities | CSIV  All facilities | REIV  All facilities | REIV  NH only | REIV  NH with skill mix |
| **Facility structure** |  |  |  |  |  |
| Not for profit | 0.049** | 0.018 | 0.020 | -0.012 | -0.009 |
|  | (0.017) | (0.019) | (0.019) | (0.046) | (0.046) |
| Nursing home | -0.038* | -0.034* | -0.037* |  |  |
|  | (0.015) | (0.016) | (0.015) |  |  |
| Dementia care | -0.031* | -0.023 | -0.029* | -0.029 | -0.026 |
|  | (0.013) | (0.013) | (0.013) | (0.022) | (0.022) |
| Size (log) | 0.026 | 0.018 | 0.007 | 0.013 | 0.016 |
|  | (0.022) | (0.022) | (0.022) | (0.033) | (0.033) |
| Occupancy rate | 0.005** | 0.005** | 0.004** | 0.006** | 0.006** |
|  | (0.001) | (0.001) | (0.001) | (0.001) | (0.001) |
| Competition (Herfindahl-Hirschman Index) | 0.272** | 0.244** | 0.247** | 0.477** | 0.472** |
|  | (0.074) | (0.077) | (0.080) | (0.120) | (0.120) |
| **Staffing** |  |  |  |  |  |
| Total staff (log) | -0.051* | -0.043* | -0.035 | -0.069 | -0.072* |
|  | (0.021) | (0.022) | (0.021) | (0.036) | (0.036) |
| Direct care staff to resident ratio | 0.263** | 0.213** | 0.202** | 0.160 | 0.160 |
|  | (0.062) | (0.065) | (0.064) | (0.111) | (0.111) |
| Direct care staff to resident ratio squared | -0.066** | -0.056** | -0.056* | -0.026 | -0.024 |
|  | (0.022) | (0.023) | (0.023) | (0.043) | (0.043) |
| Supervisor to direct care staff ratio | 0.162* | 0.130 | 0.111 | 0.194 | 0.195 |
|  | (0.075) | (0.079) | (0.060) | (0.180) | (0.181) |
| Female staff % | 0.001 | 0.001 | 0.001 | 0.0002 | 0.0017 |
|  | (0.001) | (0.001) | (0.001) | (0.0002) | (0.0013) |
| Registered nurse % |  |  |  |  | 0.094 |
|  |  |  |  |  | (0.130) |
| *Training incidence* |  |  |  |  |  |
| Dementia trained staff % | 0.001** | 0.001** | 0.001** | 0.001** | 0.001** |
|  | (0.0002) | (0.0002) | (0.0002) | (0.0004) | (0.0004) |
| Dignity/PCC trained staff % | 0.0002 | 0.0002 | 0.00005 | -0.0004 | -0.0004 |
|  | (0.0003) | (0.0003) | (0.0003) | (0.001) | (0.001) |
| *Wage* |  |  |  |  |  |
| Direct care staff hourly wage (2018 £) (log) | 0.274** | 0.834** | 0.806** | 0.945** | 0.919** |
|  | (0.082) | (0.134) | (0.138) | (0.279) | (0.275) |
|  |  |  |  |  |  |
| Local area controls | YES | YES | YES | YES | YES |
| Year | YES | YES | YES | YES | YES |
| Region | YES | YES | YES | YES | YES |
| Observations | 7,082 | 6,250 | 6,250 | 2,441 | 2,441 |
| Number of facilities | 3,652 | 3,493 | 3,493 | 1,363 | 1,363 |

Notes: CS = Cross-section; IV = instrumental variable; REIV = random effects instrumental variable; NH = Nursing homes; PCC = Person Centred Care. Robust standard errors in parentheses (clustered at facility level). All models estimated are linear probability models using OLS. Local area controls are Job Seeker’s Allowance uptake, Pension Credit uptake, Attendance Allowance uptake and average house price (log).

* p<0.05, ** p<0.01.
